# How Does Employee–Organization Relationship Affect Work Engagement and Work Well-Being of Knowledge-Based Employees?

**DOI:** 10.3389/fpsyg.2022.814324

**Published:** 2022-03-09

**Authors:** Yi Che, Jian Zhu, Huawei Huang

**Affiliations:** ^1^International College, National Institute of Development Administration, Bangkok, Thailand; ^2^Business School, University of Sanya, Sanya, China; ^3^Business School, Xiangtan University, Xiangtan, China; ^4^School of Public Administration, Xiangtan University, Xiangtan, China

**Keywords:** employee–organization relationship, need satisfaction, perceived symbiotic relationship, work engagement, work well-being, knowledge employees

## Abstract

In the employment relationship, organizational factors are the main factors that affect employee behavior. Especially for knowledge-based workers, it is even more crucial for organizations to give enough attention to their individual needs. Based on Existence, Relatedness, and Growth (ERG) theory, this study constructs a moderated mediating model to explore how the impact of the employee–organization relationship (EOR) on work engagement (WE) and work well-being (WWB) of knowledge-based employees. In this study, existence–relatedness–growth need satisfaction (GNS) is used as a mediator and the perceived symbiotic relationship is used as a moderator. Data collected from 791 knowledge-based employees in higher education institutions from more than 20 provinces and cities in China are used to test the model. The results show that (1) EOR has significant positive effects on WE and WWB. (2) Need satisfaction for relatedness partially mediates the effects of EOR on WE and WWB. (3) Need satisfaction for growth mediates the effect of EOR on WE while the mediating role of need satisfaction for growth between EOR and WWB is unsupported. (4) The mediating role of need satisfaction for the existence of EOR on both WE and WWB is unsupported. (5) The perceived symbiotic relationship moderates the relationship between EOR and WE and WWB. The findings are of theoretical significance in expanding the research field of EOR and providing a basis for organizations to implement EOR strategies.

## Introduction

With continuous breakthroughs in science and technology, knowledge has become the driving force of a new era and a decisive factor in economic and social development. In such a context, knowledge-based employees have replaced the traditional labor force and become core employees in various organizations (Liao and Wen, 2009). Hence Drucker (1992), whether knowledge-based employees perform well or did not perform well have a significant impact on the overall functioning of organizations. From the perspective of organizational human resource management practice, the main challenge is to balance the expected changes in the relationship between the organization and employees, which directly determines the individual behavior of knowledge-based employees affecting organizational performance; in particular, due to the characteristics of knowledge-based employees, organizations need to pay more attention to individual characteristics and the behavior of the group. Workplace factors contribute to both positive and negative employee outcomes. To simplify, “engagement and burnout are two poles on the workplace behavior spectrum” (Martin, 2020). On one hand, knowledge-based employee engagement will directly affect employees’ work efficiency, which decisively impacts organizational performance and customer satisfaction (Kahn, 1990). On the other hand, work is a means of earning a living and realizing self-worth for employees, so the well-being that they feel at work directly affects their level of effort. Existing research also shows that improving work well-being (WWB) is the best way to improve personal and organizational performance (Daniels and Harris, 2000; Wright and Cropanzano, 2000, 2004). Therefore, it is worth considering how to realize the alignment of interests and establish a synergistic relationship between employees and organizations.

Employee–organization relationship (EOR) is a mainstream perspective to discuss the factors that influence employee behavior within the organization in the current research. It refers to the relationship between employees and employers, including formal, informal, social, and psychological relationships in the form of the employer’s expectations about specific contributions from employees and the inducements offered to affect their contributions (Tsui et al., 1995, 1997). As an important aspect of the employment relationship, organizational factors have their main influence on employee engagement (Eldor and Harpaz, 2014). Therefore, existing research primarily focuses on the impact of the EOR on the employee’s attitude and behavior within the organization. Scholars divide EOR into four modes, such as organization-oriented, work-oriented, over-investment, and under-investment, and discuss the relationship between organizational trust, organizational commitment, and employee performance under different EOR models. Tsui et al. (1997) believe that compared with under-investment and work-oriented EOR, employees in organization-oriented and over-investment EOR show higher job performance; Hom et al. (2009) found that organization-oriented EOR and organizational commitment (emotional commitment) exhibit a positive relationship, whereas work-oriented EOR has a negative impact on organizational commitment. Social exchange theory that explains human behavior and the social structure of its relations (Kim and Qu, 2020) is mainly applied to discuss the mechanism between EOR and the employee’s working outcomes.

Due to the characteristics of knowledge-based employees, more attention is required for organizations to take care of the individual needs of this group. However, while most of the existing analyses focused on the impact of EOR on the behavior of employees, no enough attention has been given to knowledge-based employees. Another noticeable omission in the literature is how EOR relates to knowledge-based employees’ engagement through the mechanism of motivational perspective. Moreover, as there is not enough systematic analysis on the knowledge-based employee’s individual needs for the purpose of exploring the mechanism of the impact of EOR on employee behavior, a few studies on the mechanisms relating EOR to employee outcomes are relatively fragmented. Consequently, how EOR relates to knowledge-based employees’ WWB through their needs, satisfaction at work has yet to be explored. As scholars have noted, paying attention to well-being is not to replace or reject economic performance but to supplement economic indicators to serve governance and corporate practices more effectively (Li and Zhao, 2015).

To clarify the mechanisms of the impact of EOR on knowledge-based worker engagement and WWB, this paper utilizes a structural equation model (SEM) based on the data of 791 faculty and staff of higher education institutions in more than 20 provinces and cities in China. First, drawing on self-determination (SDT) theory (Deci and Ryan, 1995) and Existence, Relatedness, and Growth (ERG) theory (Alderfer, 1972), we further explicate the motivational mechanisms through which EOR affects knowledge-based employee engagement and WWB. According to the metatheory that is the basis of SDT, intrinsic motivation and internalization are the processes that require an active role of motivational factors. Specifically, people who experience a higher degree of satisfaction of their needs are more inclined to internalize their own value (Gagné and Deci, 2005). Relationships that satisfy human basic needs or essential conditions for psychological growth and well-being are associated with an intrinsic motivation (Deci and Ryan, 1995; Ryan and Deci, 2001). Therefore, we propose that when EOR is favorably perceived, it can best satisfy knowledge-based employee’s needs for ERG, which will further bring about positive work outcomes, including a number of positive attitudes and improved behaviors (Gagné and Deci, 2005). We then explore the mechanism by which EOR enhances engagement and WWB of knowledge-based employees through satisfying their need.

Second, we extend the model to reveal the significance of a symbiotic relationship between employees and organizations. Some EOR scholars have begun to explore a new paradigm of organizational management from the perspective that there is not only an exchange relationship between employees and organizations, but also a symbiotic relationship that requires the organization to provide clear definitions and designs to ensure that individual goals are consistent with organizational goals (Chen, 2016).

Research shows that EOR significantly improves knowledge-based employee engagement and WWB, while satisfaction of the need for relatedness partially mediates the impact of EOR on knowledge-based employee engagement and WWB. Satisfaction of the need for growth mediates the impact of EOR on knowledge-based employee engagement; however, its mediating role in EOR and knowledge-based employees’ WWB has not been verified. The mediating role of need satisfaction for growth in EOR and knowledge-based employee’s engagement as well as WWB have not been verified. Furthermore, knowledge-based employee’s perceived symbiotic relationship plays a moderating role in the impact of EOR on employee engagement and WWB.

The main contributions of this paper are as follows: First, to analyze the behavior of knowledge-based employees under the EOR model, we administered a questionnaire to 791 knowledge-based employees in universities in more than 20 provinces and cities in China to gather first-hand data on the relationship between EOR and knowledge-based employees’ engagement and WWB. We examined EOR, employee WWB, and engagement within the same framework, expanding the scope of EOR research. Second, this study enriches the field’s understanding of the mechanism of influence of EOR on knowledge-based employees’ behavior. Using the ERG theory, we explore a mediating role played by the satisfaction of ERG needs in the mentioned relationships, which provides a theoretical basis for organizational EOR strategy. Third, we use the survey data to empirically test a moderating role of the perceived symbiotic relationship in the impact of EOR on the behavior of knowledge-based employees, which provides a possible new research perspective for current EOR research that is based on the social exchange theory. Therefore, this study contributes to the EOR literature by introducing a new perspective at the individual level and exploring the boundary conditions of the effects of EOR on employee work engagement (WE) and WWB. Based on our findings, we give recommendations for ways to improve knowledge-based employee’s engagement and WWB in Chinese higher education institutions.

## Literature Review and Hypotheses

### The Relationship Between Employee–Organization Relationship and Work Engagement

Employee–organization relationship consists of two primary components: organization inducements (OIs) and expected contributions (ECs; March and Simon, 1958). Social exchange theory provides a theoretical underpinning for the relationship between EOR and employee WE, indicating that EOR influences employees’ work attitude and behavior (Wright and Cropanzano, 2004). Specifically, employees who feel they are favorably treated by the organization will reciprocate with desired behaviors (Blau, 1964).

In parallel, training, salary, welfare, and other developmental and material incentives provided by an organization correlate with the degree of employee engagement. Generally, when the value of these human resource indicators are high, employees’ passion for working can be increased, enhancing employee engagement (Zhang and Liu, 2007). Career planning and other human resource management strategies of an organization also significantly affect employee engagement (Yang, 2009). When the organization provides incentives and expects employees to make contributions, employees demonstrate higher standards of job performance (Jiang and Dai, 2012). Incentives have a positive effect on employees’ engagement with the organization and their work (Xi et al., 2018). Albro and McElfresh (2021) study on the workplace experiences of academic librarians during COVID-19 and has a potential impact for libraries considering the effects of workplace modifications on employee experience and retention. Synthesizing the abovementioned literature studies, we propose the following hypotheses:

*H1a1*: Organizational inducements affect employees’ WE.
*H1a2*: ECs affect employees’ WE.

### The Relationship Between Employee–Organization Relationship and Work Well-Being

According to the inducement-contribution view (March and Simon, 1958), in the EOR, when the two dimensions of organizational inducement and ECs are high, employees often perceive that their organization has created a harmonious labor–management climate (Li and Zhao, 2015). On one hand, organizational inducements were found to be a main factor predicting WWB: salary and benefits, comprehensive compensation, organizational support, supportive human resource management, and high-performance work systems (Graham and Pettinato, 2002b; Wood and Menezes, 2011; Qiu and Zhou, 2012; Zhu, 2013; Chen et al., 2018). On the other hand, when employees feel that they are expected to make greater contributions or are acknowledged by the organization, their positive work mood can be significantly improved (Martens et al., 1990). Perceived organizational support had a significant relationship with WWB. In other words, this means that supportive work environments should foster frontline employees’ psychological empowerment, as well as their WE and service-oriented organizational citizenship behaviors (Meira and Hancer, 2021), using creative means to develop the EOR when organizations cannot increase pay will increase WWB (Jolly et al., 2021). Thus, we propose the following hypotheses:

*H2a1*: OIs affect employees’ WWB.
*H2a2*: ECs affect employees’ WWB.

### The Effects of Employee–Organization Relationship on Existence, Relatedness, and Growth Need Satisfaction

According to the model of individual–organization fit (Caplan, 1987), individual behavior is affected by an interaction between individuals and their environmental context. Accordingly, the feeling of fit generated by an interaction between the individual and the environment has a positive impact on the individual as well as the organization. The “Inducement-Expectation” theory (Barnard, 1938), from which EOR was derived, defines inducement as the organization’s incentives to employees and the expectations placed on employees to contribute to the development of the organization. In other words, a prerequisite for the organization to obtain the expected employee contributions is to provide sufficient incentives. Therefore, whether an incentive is effective depending on whether it meets the needs of employees (Caplan, 1987) and the degree of satisfaction that employees perceive. When an organization understands the needs of employees and provides incentives that meet those needs, it can effectively increase employees’ work motivation.

For employees, an exchange between themselves and the organization means that they obtain various forms of incentives in exchange for the actual and ECs they bring to the organization (Shaw et al., 2013). Accordingly, employees hope that their efforts will be compensated with basic economic returns that meet their material needs and developmental returns that meet their personal growth needs. At the same time, employees also hope to establish positive interactions with the organization, receive acknowledgment, and establish good interpersonal relationships. Reasonable organization for employees not only speaks, keeps the promise, and treats employees fair and just, respects the choice and behavior, such an organization is the ideal organization type, so the staff will have a strong sense of identity and sense of belonging to the organization, willing to pay more effort and efforts for the organization, so as to enhance the employees to the organization of civic behavior (Xia et al., 2020). To this end, employees will perceive whether the material returns and developmental returns provided by the organization can meet ERG needs to generate responsive work motivations and behaviors. Based on this analysis, our study proposes the following hypotheses:

*H1b1*: OIs affect employees’ need for existence.
*H1b2*: OIs affect employees’ need for relatedness.
*H1b3*: OIs affect employees’ need for growth.
*H1b4*: ECs affect employees’ need for existence.
*H1b5*: ECs affect employees’ need for relatedness.
*H1b6*: ECs affect employees’ need for growth.

### Mediating Role of Need Satisfaction

If the organization provides a rich and long-term inducement in the EOR, employees tend to think that they are valued, trusted, and treated well (Hom et al., 2009), and thus will provide rewards for the organization according to the social exchange theory. Several reasons may account for this phenomenon. Firstly, a reasonable salary is necessary to meet an individual’s basic or existence needs (Alderfer, 1972), and salaries and benefits have an important effect on the WE of knowledge-based employees (Lester and Lester, 2001; Nguyen, and Chi, 2014). There is a positive correlation found between salary level satisfaction and positive work outcomes (Bordia and Blau, 2003), such that the satisfaction of existence needs will have a positive effect on positive work outcomes. Secondly, as explained earlier in the discussion of the characteristics of knowledge-based employees’ needs, interpersonal relationships, teamwork, and other factors are the confirmed necessities for knowledge-based employees (Wen and Wu, 2003). When people experience a communication and mutual understanding with others to establish intimate relationships and gain respect, their relatedness needs are satisfied (Deci and Ryan, 2000), and some scholars have shown that this can improve employee engagement (Gorman, 2003). Employees also require paths for personal growth and development (Alderfer, 1972). Organizations that fail to meet these needs will lose key employees. Organizational rewards help meet the need for existence, but employees also desire to develop their abilities in the organization, through which their need for growth and development is satisfied (Alderfer, 1972). Promotion opportunities show positive correlations with knowledge-based employees’ worker engagement (Cordero et al., 1994), while career responsibilities, skill diversity, and job feedback have the most significant effect on engagement (Kochanski and Ledford, 2016).

Based on the abovementioned discussion, we expect that employees’ positive need satisfaction triggered by both OIs and ECs will be related to their WE, and the following hypotheses is proposed:

*H1c1*: Existence need satisfaction (ENS) affect employees’ WE.
*H1c2*: Relatedness need satisfaction (RNS) affects employees’ WE.
*H1c3*: Growth need satisfaction (GNS) affects employees’ WE.
*H1c4*: ENS mediates the relationship between OI and employees’ WE.
*H1c5*: RNS mediates the relationship between OI and employees’ WE.
*H1c6*: GNS mediates the relationship between OI and employees’ WE.
*H1c7*: ENS mediates the relationship between EC and employees’ WE.
*H1c8*: RNS mediates the relationship between EC and employees’ WE.
*H1c9*: GNS mediates the relationship between EC and employees’ WE.

The need satisfaction theory believes that an individual’s perception of well-being comes from the satisfaction of needs. In line with ERG theory, when all three needs are satisfied, employees also perceive that they have achieved well-being at work (Kinnafick et al., 2013).

A previous study found a positive relationship between well-being and income levels. That is, the higher the income, the higher level the well-being. This conclusion is confirmed in both developed countries such as the United States and 17 Latin American developing countries (Easterlin, 2001; Graham and Pettinato, 2002a).

A few studies have also shown that meeting relatedness needs is positively correlated with an improvement in well-being (for example, Patrick et al., 2007; Boezeman and Ellemers, 2009; Milyavskaya and Koestner, 2011); employees’ satisfaction with the needs of relatedness is also closely related to job satisfaction (Broeck et al., 2010).

The improvement of self-value and the realization of goals will increase people’s perception of well-being (Edwards and Rothbard, 1999). When the relationship between personal development and well-being and health is measured in terms of the employee’s effort to improve their ability to work, the ability to learn new skills related to work, and the willingness to seek more learning opportunities due to work needs, the more time and energy employees spend on personal growth, and the higher level the perceived well-being (Arnetz and Wiholm, 1997). Based on the abovementioned analysis, the following hypotheses are made:

*H2b1*: ENS affects employees’ WWB.
*H2b2*: RNS affects employees’ WWB.
*H2b3*: GNS affects employees’ WWB.
*H2b4*: ENS mediates the relationship between OI and employees’ WWB.
*H2b5*: RNS mediates the relationship between OI and employees’ WWB.
*H2b6*: GNS mediates the relationship between OI and employees’ WWB.
*H2b7*: ENS mediates the relationship between EC and employees’ WWB.
*H2b8*: RNS mediates the relationship between EC and employees’ WWB.
*H2b9*: GNS mediates the relationship between EC and employees’ WWB.

### Moderating the Impact of Perceived Symbiotic Relationship

Symbiosis originated in biology and is defined as the mutually beneficial physical association of different organisms (Oulhen et al., 2016). The expansion of symbiosis theory in the fields of economics, sociology, and business management arose from the symbiosis thought (Hawken, 1994; Sen, 1996). Scholars have widely used the symbiosis theory in the field of business ecology and have achieved several interesting results (Rao, 2010). It has also been used to discuss enterprise clusters from a micro perspective, significantly expanding the scope of application of the symbiosis theory (Cheng, 2012; Rao, 2010).

Although the application of the symbiosis theory in each research area has its own merits, its original core concepts include interdependence, coexistence, and co-evolution between symbiotic units. The construct shares many similarities with communal relationships wherein individuals are dependent on each other through coexisting and sharing. To date, research on the symbiosis theory has rarely been applied to the field of organizational behavior, but some scholars have shown that in social sciences, the mutual connections and mutual influence derived from symbiotic relationships in biology also exist between people, among companies upstream and downstream in the supply chain, and in enterprise alliances. Further, on the individual level, there is also a symbiotic relationship between husband and wife, employers and employees, parents and children, and teachers and students (Yang, 2010). Some scholars have even proposed that relationships between employees and organizations have evolved from the leader-subordinate model into the symbiotic model (Chen, 2016; Zhai and Wang, 2017).

Knowledge-based employees are valuable contributors to the organization’s development and success and thus employers should cultivate a symbiotic employment relationship on the premise of mutual benefit (Etzioni, 1961; Tsui et al., 1995). The effectiveness of the EOR will be affected by situational factors such as relationship to the organization, leadership, work type, and individual traits (Zhao, 2011). The latter includes individual employees’ perceptions, values, awareness, etc. The continuous investment and contribution of both the employer and employee can maintain EOR and promote the evolution of a symbiotic system. Therefore, we posit that employees’ perception of their level of interdependence and mutual development within the organization plays an important role in the relationship between OI and WE, as well as between EC and WE. This study thus proposes the following hypotheses:

*H1d1*: The perceived symbiotic relationship moderates the relationship between OI and employees’ WE such that the engagement levels are higher for employees with an increase of the perceived symbiotic relationship.
*H1d2*: The perceived symbiotic relationship moderates the relationship between EC and employees’ WE such that the engagement levels are higher for employees with an increase of the perceived symbiotic relationship.

According to previous studies of the EOR, employees’ perceptions of organizational inducements and ECs affect their perceptions of whether the labor–management relationship is harmonious (Li and Zhao, 2015), and such a harmony will improve employees’ work experiences and effectiveness (Chen et al., 2013). Thus, it can be inferred that EOR perceptions will impact employees’ experience at work and their degree of self-value realization. For employees, the degrees of interdependence, inter-influence, and mutual development that they perceive are different; therefore, employees’ WWB will be different.

In summary, a symbiotic relationship is helpful in the formation of harmonious labor–management relationships; the OIs and ECs, which are considered as an employment relationship model, will affect whether employees perceive a harmonious labor–management relationship with the organization. In other words, the extent of symbiotic relationship between employees and the organization will affect their perception of EOR on job well-being. Therefore, this study proposes the following hypotheses:

*H2c1:* The perceived symbiotic relationship moderates the relationship between OI and employees’ WWB such that the well-being is stronger for employees with increasing perceived symbiotic relationship.

*H2c2:* The perceived symbiotic relationship moderates the relationship between EC and employees’ WWB such that the well-being is stronger for employees with increasing perceived symbiotic relationship.

**FIGURE 1:**
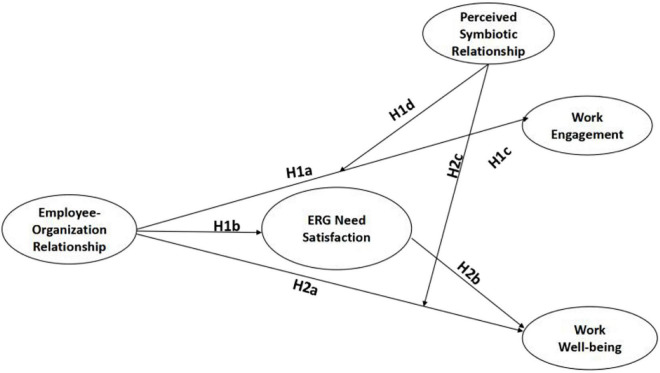
Theoretical model.

The theoretical model used in this study is shown in Figure 1.

## Research Method

### Sample and Procedure

The respondents of this research included teaching and research faculty and managerial staff in colleges and universities from more than 20 provinces in China. These personnel are considered as typical knowledge-based employees as defined by Liu et al. (2018). A total of 1,170 questionnaires were distributed, and 830 responses were retrieved electronically. Of these, surveys with an answer time of less than 360 s were deleted, leaving 791 validated questionnaires obtained for this research (the recovery rate of 85.6%). Table 1 shows the sample description.

**TABLE 1:** Sample description.

Variables	Categories	Frequency	Valid percentage	Cumulative percentage
Age	≤25	31	3.9	3.9
	26–35	287	36.3	40.2
	36–45	294	37.2	77.4
	>45	179	22.6	100
Gender	Male	289	36.5	36.5
	Female	502	63.5	100
Education	Bachelor	237	30	30
	Master	443	56	86
	Ph.D and +	111	14	100
Professional title	Lecturer and –	532	67.3	67.3
	Assoc. Prof.	165	20.9	88.1
	Prof.	94	11.9	100
Working years with current employment	<3	122	15.4	15.4
	3–7	175	22.1	37.5
	7–10	134	16.9	54.5
	>10	360	45.5	100
Total working years	<3	113	14.3	14.3
	3–7	155	19.6	33.9
	7–10	119	15	48.9
	>10	404	51.1	100
Job function	Faculty	454	57.4	57.4
	Staff	337	42.6	100.0

### Measurement

Scales are selected by consulting the literature and combining the operative research variables. For survey instruments that were originally constructed in English and translated into Chinese, we followed the recommended back translation procedures (Brislin, 1980). Unless otherwise indicated, response options ranged from (1) “strongly disagree” to (7) “strongly agree.”

We used Jia et al.’s (2014) 27-item scale to assess EOR. Sample survey statements include: “My organization provides competitive salaries”; “My organization emphasizes employee career development”; “My organization expects employees to fulfill the job inside and outside”; and “My organization expects employees to take initiative to make constructive suggestions.”

Work engagement was measured by using the simplified version of the Utrecht Work Engagement Scale (UWES) with nine items compiled by Schaufeli and Bakker (2003). Sample statements include: “At my job, I feel strong and vigorous” and “I am proud of the work that I do.”

We measured WWB with the six-item scale developed by Zheng et al. (2015). One of the sample statements is “In general, I feel fairly satisfied with my present job.”

This study refers to the methodology of Li et al. (2016) and selects three variables: salary satisfaction, work relationship, and career growth, to represent the need for ERG of knowledge-based employees, respectively. The measurement of salary satisfaction is adapted from Judge’s salary satisfaction scale (Judge, 1993), a sample statement is “I am satisfied with my current salary.” The measurement of need for relatedness adopts the relational demand dimension of the basic demand scale developed by Broeck et al. (2010). There are six items in total, a typical question is “At work, I can talk with people about things that really matter to me.” The measurement of need for growth adopts the scale of Lee and Bruvold (2003) with a total of nine items, one typical item is “the organization provides employees with career consultation and planning guidance.”

To assess the perceived symbiotic relationship, the communal relationship scale developed by Johnson and Grimm (2010), in combination with components in the two constructs of organizational citizenship behavior and intention to stay.

### Data Analysis

In this study, psychological isolation was used to prevent common method variance (CMV), and each scale in the questionnaire was an independent part (Podsakoff et al., 2003). Data analysis methods include reliability test, discriminant validity test, convergent validity test, correlation coefficient test, and regression analyses.

## Results

### Reliability Analysis

In this study, Statistical Product and Service Solution (SPSS) was used to test the reliability of the scales. The test results show that the Cronbach’s α for OI and EC is 0.974 and 0.967, respectively; ERG need satisfaction is 0.956; WE is 0.959; WWB is 0.943; and the perceived symbiotic relationship scale is 0.973.

### Model Goodness-of-Fit

We conducted a confirmatory factor analysis (CFA) to determine the best fitting model. Because the IFI, CFI, and NFI values in the first analysis of factor structure validity did not reach the ideal standard, the index modification (MI) was executed. After modification, χ^2^/*df* = 3.208, RMSEA = 0.053 < 0.8, CFI = 0.931 > 0.9, NFI = 0.926 > 0.9, IFI = 0.931 > 0.9, PNFI = 0.809 > 0.5, PGFI = 0.707 > 0.5, where each model fitting index met or exceeded the fitting standard (Ho, 2006). Table 2 summarizes the results of the internal fitting indicators of CFA before and after modification.

**TABLE 2:** Model fit.

Fit indices	Recommended value	Original model fit	Model fit after MI
CMIN/DF(χ^2^/df)	<5.00	4.889	3.208
RMSEA	<0.08	0.070	0.053
IFI	>0.90	0.876	0.931
CFI	>0.90	0.875	0.931
NFI	>0.90	0.848	0.926
PNFI	>0.50	0.812	0.848
PCFI	>0.50	0.838	0.875

### Convergent Validity

The composite reliability is calculated from the standardized regression weights in the CFA model. CR > 0.6 indicates that the latent variables have good combination reliability (Diamantopoulos and Siguaw, 2000). The calculation results showed that the composite reliability of OI, EC, need for existence satisfaction, need for relatedness satisfaction, need for growth satisfaction, WE, and WWB are 0.971, 0.959, 0.931, 0.930, 0.957, 0.958, and 0.939, respectively; all the values are > 0.6, indicating that the latent variables in the model have a good consistency.

According to the recommendations of Fornell and Larcker (1981), this study used the average variable extraction (AVE) value to check the aggregate validity of each scale; all values are between 0.68–0.77 and all >0.5, which proved that each scale has good aggregation validity.

### Discriminant Validity and Correlation Analyses

The CFA was used to compare the AVE value and the square of the correlation coefficient between the variables. The results are shown in Table 3. Taking OI as an example, the square of the Pearson correlation coefficient between OI and other variables is between 0.27 and 0.62, AVE = 0.87, which is greater than the Pearson correlation coefficient square value. Thus it can be seen that OI scale has good discriminative validity.

**TABLE 3:** Discriminant validity analysis.

	OI	EC	ENS	RNS	GNS	WE	WWB
OI	0.870						
EC	0.272**	0.825					
ENS	0.611**	0.183**	0.880				
RNS	0.414**	0.345**	0.448**	0.831			
GNS	0.537**	0.236**	0.652**	0.555**	0.846		
WE	0.626**	0.410**	0.538**	0.520**	0.538**	0.847	
WWB	0.530**	0.344**	0.454**	0.541**	0.481**	0.676**	0.850

**The correlation is significant at the level of 0.01 (two-tailed), and the diagonal is the mean square root of average variable extraction (AVE).

*OI, organization inducement; EC, expected contribution; ENS, existence need satisfaction; RNS, relatedness need satisfaction; GNS, growth need satisfaction; WE, work engagement; WWB, work well-being.*

### Hypothesis Test

The SEM is used to test the relationships among variables of OI, EC, ENS, RNS, GNS, and WE, as well as WWB, by using AMOS 23.0 to verify the hypotheses in relation to direct effects and mediating roles.

(1) Testing the relationship between OI, EC, ENS, RNS, GNS, WE, and WWB (see Table 4).
Hypotheses H1a1 and H1a2 proposed that OIs and ECs affect WE. As shown in Table 4, the effects of two variables on WE are β = 0.378, *p* < 0.001; β = 0.200, *p* < 0.001. Hypotheses H1a1 and H1a2 are thus supported.
Hypotheses H1b1, H1b2, and H1b3 proposed that OIs affect ENS, RNS, and GNS, respectively, while EC proposed in H1b4, H1b5, and H1b6 affects ENS, RNS, and GNS, respectively. As shown in Table 4, the β of OI on ENS is 0.638, *p* < 0.001, indicating that OI has a significant positive impact on ENS; the β of EC on ENS is 0.023, *p* = 0.458 > 0.05, and the coefficient is negative, indicating that the EC has no significant negative effect on ENS; the β’s of OI and EC on RNS are 0.387, *p* < 0.001 and 0.244, *p* < 0.001, indicating that OI and EC have a significant positive impact on RNS; the β of OI and EC on GNS are 0.557, *p* < 0.001 and 0.086, *p* < 0.001, respectively, indicating that OI and EC have a significant positive impact on GNS. Hypotheses H1b1, H1b2, H1b3, H1b5, and H1b6 are supported, and H1b4 is rejected.
Hypotheses H2a1 and H2a2 proposed that OIs and ECs affect WWB. As shown in Table 4, the effects of these two variables on WWB are β = 0.304, *p* < 0.001; β = 0.124, *p* < 0.001, therefore hypotheses H2a1 and H2a2 are supported.
Hypotheses H1c1, H1c2, and H1c3 proposed that ENS, RNS, and GNS affect WE. As shown in Table 4, the β values of ENS on WE, RNS on WE and GNS on WE are β = 0.123, *p* < 0.001; β = 0.219, *p* < 0.001; β = 0.101, *p* = 0.002 < 0.05, thus hypotheses H1c1, H1c2, and H1c3 are supported.
Hypotheses H2b1, H2b2, and H2b3 proposed that ENS, RNS, and GNS affect WWB. As shown in Table 4, the β values of ENS on WWB, RNS on WWB, and GNS on WWB are β = 0.081, *p* = 0.033 < 0.05; β = 0.329, *p* < 0.001; β = 0.080, *p* = 0.026 < 0.05, thus H2b1, H2b2, and H2b3 are supported.
(2) Testing a mediating role of ENS, RNS, and GNS in the relationship between independent and dependent variables.

**TABLE 4:** Path analysis.

Path			Unstandard regression weight	*SE*	C.R.	*P*	Standard regression weight
ENS	<–	OI	0.714	0.040	18.003	***	0.638
RNS	<–	OI	0.371	0.035	10.628	***	0.387
GNS	< —	OI	0.611	0.040	15.372	***	0.557
GNS	< —	EC	0.122	0.046	2.664	0.008	0.086
RNS	< —	EC	0.305	0.044	6.928	***	0.244
ENS	< —	EC	–0.033	0.045	–0.741	**0.458**	**–0.023**
WE	< —	OI	0.315	0.036	8.724	***	0.378
WWB	< —	EC	0.142	0.036	3.960	***	0.124
WE	< —	EC	0.217	0.031	6.928	***	0.200
WE	< —	ENS	0.092	0.025	3.607	***	0.123
WWB	< —	ENS	0.064	0.030	2.136	0.033	0.081
WE	< —	RNS	0.077	0.027	6.964	***	0.219
WWB	< —	RNS	0.268	0.033	9.177	***	0.329
WWB	< —	GNS	0.190	0.029	2.231	0.026	0.080
WE	< —	GNS	0.302	0.024	3.130	0.002	0.101
WWB	< —	OI	0.063	0.041	6.473	***	0.304

***p < 0.001.

*The bold value highlights that the regression weight is negative.*

To test a mediating effect of ENS, RNS, and GNS in the relationship between OI and WE, EC and WE, OI and WWB, EC and WWB, further analyses were performed to confirm the magnitude and statistical significance of the indirect effects. Specifically, the bootstrap CIs method with 2,000 iterations was applied, adopting the recommendations of Preacher and Hayes (2008). The test results are presented in Table 5.

**TABLE 5:** Bootstrap test results of total effect, direct effect and indirect effect in the model.

	CIs bias Corrected			
Path	Standardized estimate	*SE*	Lower confidence level	Upper confidence level
	Total Effect			
OI-WE	0.497	0.035	0.425	0.570
OI-WWB	0.464	0.037	0.388	0.551
EC-WE	0.282	0.044	0.196	0.382
EC-WWB	0.240	0.053	0.134	0.3444

	**Indirect Effect**			

OI-ERG-WE	0.183	0.037	0.114	0.251
OI-ENS-WE	0.065	0.029	**–0.004**	**0.127**
OI-RNS-WE	0.071	0.02	0.037	0.119
OI-GNS-WE	0.047	0.023	0.009	0.101
OI-ERG-WWB	0.196	0.04	0.106	0.277
OI-ENS-WWB	0.045	0.031	**–0.017**	**0.097**
OI-RNS-WWB	0.112	0.026	0.063	0.169
OI-GNS-WWB	0.039	0.027	**–0.010**	**0.109**
EC-ERG-WE	0.064	0.017	0.030	0.098
EC-ENS-WE	–0.003	0.007	**–0.022**	**0.006**
EC-RNS-WE	0.058	0.014	0.034	0.088
EC-GNS-WE	0.009	0.006	0.002	0.030
EC-ERG-WWB	0.098	0.024	0.059	0.147
EC-ENS-WWB	–0.002	0.005	**–0.020**	**0.003**
EC-RNS-WWB	0.092	0.021	0.057	0.135
EC-GNS-WWB	0.008	0.006	**–0.001**	**0.024**

	**Direct Effect**			

OI-WE	0.268	0.052	0.166	0.372
OI-WWB	0.315	0.049	0.227	0.417
EC-WE	0.142	0.051	0.038	0.265
EC-WWB	0.217	0.045	0.127	0.307

*OI, organization inducement; EC, expected contribution; ENS, existence need satisfaction; RNS, relatedness need satisfaction; GNS, growth need satisfaction; WE, work engagement; WWB, work well-being.*

*The bold value highlighted that there is a “0” between the lower confidence level and the upper confidence level.*

In the study, Hypotheses H1c4, H1c5, and H1c6 proposed a mediating effect of ENS, RNS, and GNS in the relationship between OI and WE; H1c7 and H1c8, H1c9 proposed a mediating effect of ENS, RNS, and GNS in the relationship between OI and WE. As shown in Table 5, H1c5 and H1c6 are supported and H1c4 is rejected, and H1c8 and H1c9 are supported while H1c7 is rejected.

(3) Testing a moderating impact of the perceived symbiotic relationship in the relationship between independence and dependence.

In this study, H1d1 and H1d2 proposed a moderating effect in the relationship between OI and WE, and EC and WE. H2c1 and H2c2 proposed a moderating effect in the relationship between OI and WWB, and EC and WWB.

According to the recommendations of Baron and Kenny (1986), a hierarchical regression analysis was used to verify moderating effects, and the conditions for the existence of a moderating effect are as follows: first, the independent variable significantly affects the dependent variable; second, a moderating variable significantly affects the dependent variable; and third, the product of the independent variable and a moderating variable significantly affects the dependent variable. The study followed the abovementioned steps to test the moderating effects of the perceived symbiotic relationship.

**FIGURE 2:**
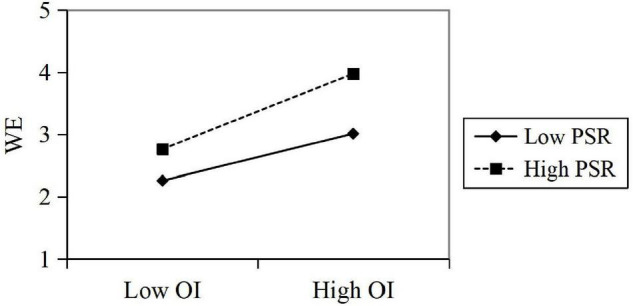
A moderating effect of PSR in organization inducement (OI) predicting work engagement (WE).

The test results showed that OI has a significant positive impact on WE (β = 0.626, *t* = 22.524, *p* < 0.05); the interaction of OI and perceived symbiotic relationship (PSR) has a significant effect on WE (β = 0.116, *t* = 4.451, *p* < 0.05), and PSR has a significant positive effect on WE (β = 0.36, *t* = 13.072, *p* < 0.05). In summary, it can be seen that the perceived symbiotic relationship has a positive moderating effect in predicting the influence of OI on employees’ WE. *R*^2^ increased by 0.109, indicating that with a moderating effect of the perceived symbiotic relationship, the explanatory power of OI on employee WE significantly increased by 10.9%. Then, the regressions are calculated in the corresponding regression equation, and the moderating effect diagram is drawn according to the regression coefficient (Figure 2).

**FIGURE 3:**
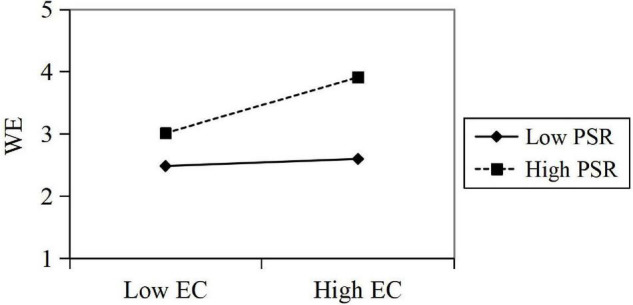
Moderating effect of PSR in expected contribution’s (EC’s) predicting WE.

The test results showed that EC has a significant positive impact on WE (β = 0.41, *t* = 12.619, *p* < 0.05); the interaction of OI and PSR has a significant effect on WE (β = 0.19, *t* = 6.474, *p* < 0.0), and PSR has a significant positive effect on WE (β = 0.46, *t* = 14.123, *p* < 0.05). In summary, it can be seen that the perceived symbiotic relationship has a positive moderating effect in predicting the influence of EC on employees’ WE. *R*^2^ increased by 0.187, indicating that with a moderating effect of the perceived symbiotic relationship, the explanatory power of EC on employee WE significantly increased by 18.7%. Then, the regressions are calculated in the corresponding regression equation, and the moderating effect diagram is drawn according to the regression coefficient (Figure 3).

**FIGURE 4:**
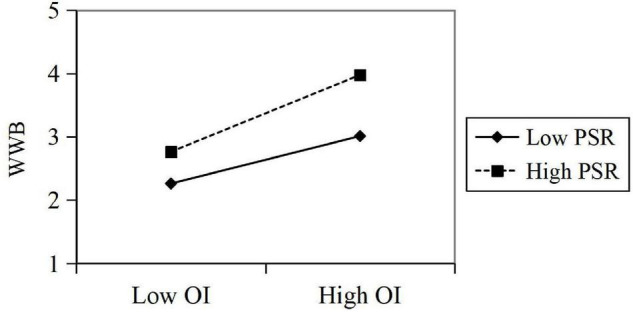
A moderating effect of PSR in OI’s predicting work well-being (WWB).

In predicting WWB, the test results showed that OI has a significant positive impact on WWB (β = 0.53, *t* = 17.545, *p* < 0.005); the interaction between OI and PSR has a significant effect on WWB (β = 0.10, *t* = 8.319, *p* < 0.05), and PSR has a significant positive effect on WWB (β = 0.26, *t* = 3.335, *p* < 0.05). In summary, these results show that the perceived symbiotic relationship has a positive moderating effect in predicting the influence of OI on employees’ WWB. *R*^2^ increased by 0.058, indicating that with a moderating effect of the perceived symbiotic relationship, the explanatory power of OI on employee WE significantly increased by 5.8%. Then, the regressions are calculated in the corresponding regression equation, and the moderating effect diagram is drawn according to the regression coefficient (Figure 4).

**FIGURE 5:**
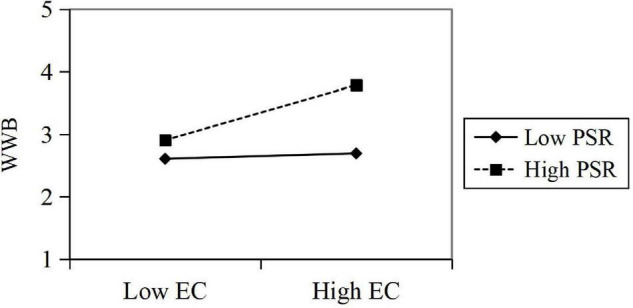
Moderating effect of PSR in EC’s predicting WWB.

The test results showed that EC has a significant positive impact on WWB (β = 0.34, *t* = 10.283, *p* < 0.05); the interaction of EC and PSR has a significant effect on WWB (β = 0.19, *t* = 6.013, *p* < 0.05), and PSR has a significant positive effect on WWB (β = 0.34, *t* = 9.82, *p* < 0.05). In summary, the data show that the perceived symbiotic relationship has a positive moderating effect in predicting the influence of EC on employees’ WWB. *R*^2^ increased by 0.120, indicating that with a moderating effect of the perceived symbiotic relationship, the explanatory power of EC on employee WWB significantly increased by 12%. Then, the regressions are calculated in the corresponding regression equation, and the moderating effect diagram is drawn according to the regression coefficient (Figure 5).

## Discussion and Conclusion

This study tests the effects of EOR on knowledge-based employees’ WE and well-being and then explores the effect mechanism from a motivational perspective. We also propose that employees’ perceived symbiotic relationship predicts employees’ WE and WWB. For the hypothesis, all proposed direct effects have been confirmed (H1a, H1a2, H1b, H1b2, H1b3, H1b5, H1b6, H1c1, H1c2, H1c3; H2a1, H2a2, H2b1, H2b2, H2b3) by a path analysis except for H1b4, the effect of EC on ENS.

The confirmed hypotheses are consistent with research conclusions of some previous scholars, such as: OIs have a significant impact on engagement (Xi et al., 2018); employees are more motivated and satisfied with higher incentives and high ECs (Tsui et al., 1997); and different organizational incentives and expectations perceived by employees affect employees’ WWB (Li and Zhao, 2015).

Hypothesis H1b4, however, is not supported as the hypothesis testing sign is negative. We understand that this may be due to the fact that the object of this research is knowledge-based employees. For these people, their ENS (in terms of salary, benefits, and training for career development) is only one aspect of their overall needs. ECs positively affect their need for relatedness such as being respected and acknowledged, and need for growth such as acquiring new knowledge and skills. However, EC does not have a significant negative impact on their need for existence. This finding is in line with what the ERG theory argues that for some people, at the same time, there may be more than one need that plays a part (Alderfer, 1969).

For the proposed mediating effects, the roles of related need satisfaction in all the relationships between OI and WE, EC and WE, OI and WWB, and EC and WWB are verified. These test results are consistent with the conclusions of few previous research. For example, meeting employees’ need for relatedness can increase employee engagement (Gorman, 2003); meeting need for relatedness is positively correlated with well-being (Patrick et al., 2007; Boezeman and Ellemers, 2009; Baccaro and Howell, 2011; Milyavskaya and Koestner, 2011).

The study results showed that if knowledge-based employees perceive that organizations value their suggestions for work, entrust them with full authority within their responsibilities, fully respect their personal dignity, encourage them to participate in the decision-making, etc., they will feel more respect and trust from the organization. As a result, they feel that work is more meaningful, and they are more dedicated to work.

Our study shows that GNS has a mediating effect on the relationship between OIs and ECs and knowledge-based employees’ engagement, consistent with previous research. For example, job responsibilities, skill diversity, and job feedback have the most significant impact on employee engagement (Kochanski and Ledford, 2016). We also found that GNS does not play a mediating role in the relationship between OIs and ECs to WWB of knowledge-based employees. The conclusions of this study may be due to the two factors: on one hand, if the three needs of employees are satisfied, the individual will sense that working creates happiness (Shaw et al., 2013); as far as knowledge-based employees are concerned, the balanced satisfaction of the three needs helps them improve their WWB. On the other hand, WWB may also be affected by other factors such as work-family balance.

The results of this study also showed that ENS does not play a mediating role in the influence of OIs on knowledge-based employees’ WE, and neither on their WWB. It does not have any mediating effect on the impact of ECs on the engagement or WWB. These research results show that for knowledge-based employees, salary and benefits may have an impact on their engagement under certain conditions although providing them with higher salaries and benefits alone is not enough to motivate them to have better performance or greater enthusiasm on their work. Higher income is not enough to make them feel the dignity and stronger reputation that work can bring them.

Similarly, for knowledge-based workers, getting higher salaries and benefits alone is not sufficient to improve their well-being because other factors, such as satisfaction with their work, feeling the joy of work, and feeling that work is meaningful, are also critical. These findings align with the need characteristics of knowledge-based employees that the three ERG types of need are important.

Perhaps the most significant finding in the study concerns a moderating impact of the perceived symbiotic relationship, where all the four proposed moderating impacts were verified. The analysis showed that when knowledge-based employees perceive the organization’s emphasis on their work suggestions, fully authorize them within the scope of their duties, provide them with the knowledge and skill training needed for their work ability improvement and career development, fully respect their personal dignity, and encourage them to participate in the decision-making of the organizations, they tend to work harder and feel happier at work. With an increase in the abovementioned sentiments, both the organization’s investment and expectations will have a stronger impact on knowledge-based employees’ WE and well-being, and in turn the employees will become more enthusiastic and committed, bringing them a greater perception of being esteemed, resulting in a better overall experience and greater sense of self-realization.

### Implications and Recommendations

The research samples are from higher education institutions, where there is a high concentration of knowledge-based employees. In such organizations, when formulating human resources policies and measures, administrators should give faculty and staff full respect and robust mechanisms for communication, debugging, and feedback, such as adopting dialog-model exchange to convey the problems that arise in the work. In addition, various platforms should be built up to encourage employees to make suggestions and then earnestly adopt their constructive suggestions. Administrators should also enhance a positive and healthy organizational culture, so as to provide knowledge-based employees with beneficial work experience.

Higher education organizations would benefit from further in-depth understanding of and increased attention to the symbiotic relationship between individuals and organizations, which fosters a win–win partnership, superseding the traditional hierarchical leader–subordinate relationship. Administrators should fully realize the importance of relationships among a community with common development rather than a hierarchical structure. The finding of employees’ perceived symbiotic relationship has opened up a new perspective for viewing the employment relationship by administrators.

This symbiotic relationship view should be considered when establishing organizational policies, systems, and culture so as to achieve a balance between organizational interests and knowledge-based employees’ needs. While it is necessary to design salary plans, training plans, and democratic platforms in a fair, reasonable, and scientific manner so that employees have a sense of security and gain, it is also necessary to vigorously encourage knowledge-based employees to perform their job responsibilities and out-of-role behavior. Further, administrators need to give more attention to the artistry of management by adopting an equal and a cooperative attitude, and prioritize the career success of knowledge-based employees when implementing relevant policies.

Material investment, development investment, and ECs from employees to the organization have a consistent impact on knowledge-based employees’ engagement and WWB. Therefore, higher education institutions should rigorously apply the characteristics of knowledge talents and implement plans from the two aspects: improving engagement and WWB happiness. In addition to motivating knowledge-based employees to be devoted and committed to work, employers can also facilitate their increased positive experiences and value at work. For example, while motivating them to produce scientific research outcomes, the organization also needs to focus on their exploration of research interests.

### Limitations and Future Research

This study selected higher education institutions with a relatively concentrated population of knowledge-based employees. Although this group is well represented in universities and colleges to make the conclusions more generalizable, future survey research should also be conducted in other types of organizations.

This study provides an initial demonstration of the perceived symbiotic relationship construct. Although it may theoretically provide new perspectives in understanding employee–organizational relationships, the measurement of this variable uses an indirect measurement, a combination of related constructs (Sun et al., 2016). Future research may consider employing more accurate methods or developing a standardized scale for the perceived symbiotic relationship.

This research studies the effect and mechanism of EOR from the perspective of employees’ perception. Because the EOR reflects the human resource management strategy and practices implemented by the enterprise, future research may launch cross-level EOR studies from both the perspectives of employers and employees. Further research regarding the consistency and differences of various EORs is needed to identify different effects and influences that are manifested in these relationships.

## Data Availability Statement

The original contributions presented in the study are included in the article/supplementary material, further inquiries can be directed to the corresponding author/s.

## Ethics Statement

The studies involving human participants were reviewed and approved by the institutional review board in the National Institute of Development Administration in Thailand. The patients/participants provided their written informed consent to participate in this study.

## Author Contributions

YC developed the theoretical model, collected the data, and wrote the manuscript. JZ was responsible for an empirical analysis. HH participated in manuscript writing. All authors contributed to the article and approved the submitted version.

## Conflict of Interest

The authors declare that the research was conducted in the absence of any commercial or financial relationships that could be construed as a potential conflict of interest.

## Publisher’s Note

All claims expressed in this article are solely those of the authors and do not necessarily represent those of their affiliated organizations, or those of the publisher, the editors and the reviewers. Any product that may be evaluated in this article, or claim that may be made by its manufacturer, is not guaranteed or endorsed by the publisher.
